# A randomised controlled trial of the effectiveness of a breastfeeding training DVD on improving breastfeeding knowledge and confidence among healthcare professionals in China

**DOI:** 10.1186/s12884-018-1709-1

**Published:** 2018-03-27

**Authors:** Yuan Ying Ma, Louise L. Wallace, Li Qian Qiu, Joanna Kosmala-Anderson, Naomi Bartle

**Affiliations:** 1 Women’s hospital, School of Medicine, Zhejiang University, Xueshi Road 1#, Shangchen District, Hangzhou, Zhejiang Province China; 2 The Open University, Walton Hall, Milton Keynes, MK7 6AA UK; 3 University of Cambridge, Trinity Lane, Cambridge, CB2 1TN UK; 4 Coventry University, Priory Street, Coventry, CV1 5FB UK

**Keywords:** Breastfeeding, DVD, Positioning, Attachment, Hand expression, Training, Midwives, Nurses, Maternity services

## Abstract

**Background:**

Despite almost all babies being breastfed initially, the exclusive breastfeeding rate at six months is less than 30% in China. Improving professionals’ knowledge and practical skill is a key government strategy to increase breastfeeding rates. This study aimed to test the effectiveness of a breastfeeding DVD training method for clinicians on improving their knowledge and confidence in the breastfeeding support skills of teaching mothers Positioning and Attachment (P & A) and Hand Expression (HE).

**Methods:**

A randomised controlled trial was conducted in three hospitals in Zhejiang province, China in 2014. Participants were recruited before their routine breastfeeding training course and randomly allocated to intervention group (IG) and control group (CG). The 15 min “Breastfeeding: Essential Support Skills DVD” was the intervention for IG and a vaginal delivery DVD was used for CG. All participants completed questionnaires of job information, knowledge and confidence in the two skills before (baseline) and immediately after viewing the DVD (post DVD).

**Results:**

Out of 210 participants, 191 completed knowledge assessments before and after watching the DVD (IG *n* = 96, CG *n* = 95), with the response rate of 91.0%. At baseline, there are no significant differences in job variables, total knowledge scores and confident scores. The total knowledge score significantly increased post-DVD for IG (pre-DVD: M = 5.39, SD = 2.03; post-DVD: M = 7.74, SD = 1.71; t (95) = − 10.95, *p* < 0.01), but no significant change in total knowledge score for CG between pre- and post-DVD (pre-DVD: M = 5.67, SD = 1.70; post-DVD: M = 5.56, SD = 1.63; t (94) = 0.85). The total confidence scores were significantly higher post-DVD than pre-DVD in IG (pre-DVD: M = 66.49, SD = 11.27; post- DVD: M = 71.81, SD = 9.33; t (68) = − 4.92, *p* < 0.01), but no significant difference was seen in CG between pre- and post-DVD total confidence scores (pre-DVD: M = 68.33, SD = 11.08; post-DVD: M = 68.35, SD = 11.40; t (65) = − 0.25). Personal and job variables did not mediate these effects.

**Conclusions:**

The breastfeeding training DVD improved professionals’ knowledge and confidence of the two breastfeeding support skills. However, the effect on professionals’ practice and on breastfeeding outcomes needs to be examined in the future.

## Background

There is strong global evidence for the short and long term health benefits of breastfeeding for infants and mothers [1–7]. Breastfeeding is recommended as the healthiest way to feed an infant by the World Health Organization (WHO) and UNICEF [8]. It is estimated that globally approximately 800,000 child lives would be saved each year if all children were fed according to the recommendations of the WHO and UNICEF [http://www.who.int/features/factfiles/breastfeeding/en/]. Therefore, promoting breastfeeding and increasing breastfeeding rates has become a global strategy to improve children’s health. Many governments are implementing various interventions to achieve national breastfeeding goals. In China, the National Programme of Action for Children’s Development (NPA) has set national targets for exclusive breastfeeding rates at six months to improve children’s health and development every ten years since 1990. The new target set by NPA for 2011–2020 is to increase the exclusive breastfeeding rate for infants aged 0–6 months to over 50% [http://www.gov.cn/gongbao/content/2011/content_1927200.htm]. However, the National Health and Family Planning Commission (NHFPC) reported the latest rate of exclusive breastfeeding at six months to be 30% in rural areas and 16% in urban areas in 2014 [http://www.womenofchina.cn/womenofchina/html1/17/3045-1.htm].

Returning to work and insufficient breast milk are reported by mothers to be the main reasons for ceasing breastfeeding early according according to Xu and colleagues who reviewed breastfeeding rates and practices in China in 41 cohort and cross sectional studies published between 1990 and 2009, and included large sample sizes [9].

The cross-sectional study by Cannon indicated that the importance of intra-oral vacuum in effective and efficient milk removal during breastfeeding. Incorrect attachment affect the removal of milk due to low vacuum [10].Ineffective removal of breast milk with incorrect positioning and attachment (P & A) wi leads to an increase in the feedback inhibitor of lactation (FIL), which is produced by the lactocytes and regulates the production of breast milk. As FIL increases, breast milk production will decrease [11, 12]. In addition, poor attachment leads to sore nipples, engorgement, mastitis and poor weight gain as the tongue is not placed over the lower gum and the gums placed over the lactiferous sinuses [13, 14], which inhibits effective milk removal from the breast. Hand Expression (HE) is an essential skill for mothers who cannot breastfeed their infants directly due to their own or their baby’s illness, infants’ separation from mothers or preterm birth [15–17]. HE is also used to stimulate breast milk production when the amount of breast milk seems to be insufficient [18]. Enhancing professionals’ skills in P & A and HE are essential for supporting breastfeeding mothers, but the challenge of training many thousands of maternity clinicians across China will require new approaches to training.

P & A and HE are two essential skills to ensure mothers breastfeed successfully, and such that they are a required part of the curriculum for training all maternity staff to enable all mothers to use these skills, which forms part of the “Ten Steps” of the UNICEF Baby Friendly Hospital Initiative (BFHI) accreditation [19], [http://www.unicef.org/nutrition/index_24806.html]. The key technique of P & A when it was taught by skilled professionals antenatally was associated with increased breastfeeding rates for as long as six months after birth among 70 mothers in a small pilot study in Western Australia [20, 21]. However, two national surveys in the UK indicated that family doctors, paediatric clinicians and nursing and midwifery clinicians often believe they have inadequate knowledge and insufficient training in breastfeeding support including teaching P & A [22, 23].

In China, there have been no studies outside of our research specifically assessing clinicians’ knowledge and skill in teaching mothers P & A and HE. However, Ouyang undertook a survey to investigate the knowledge of a range of breastfeeding topics among 600 Chinese female physicians and nurses from 10 randomly selected BFHI hospitals in Hubei province in 2009 [24]. The knowledge assessment used statements which were scored true or false, and covered the benefits of breastfeeding, reliable signs of adequate breast milk, demand feeding and recommended duration of breastfeeding. The results showed most clinicians had surprisingly poor knowledge of breastfeeding, and 79.3% of clinicians had received no breastfeeding training since they began working in maternity hospitals. In 2014, a spokesman from UNICEF China said that there are a lack of skilled professionals in hospital and community servces to satisfy an emerging group of women in China who are eager to breastfeed [http://www.womenofchina.cn/womenofchina/html1/17/3045-1.htm]. Watkins and Dodgson suggested that one of the most efficient strategies for increasing breastfeeding duration and exclusive breastfeeding according to previous systematic reports was effective breastfeeding training for professionals [25]. A recent review of 1192 studies aimed to identify whether education and training programs for healthcare staff have an effect on their knowledge and attitudes about supporting breastfeeding women. However, the review was inconclusive due to low study quality. Therefore, robust research design is necessary in future research [26].

The current study was designed to assess the specific knowledge and confidence to practise the skills of P & A and HE. It also tested whether training clinicians with a short breastfeeding DVD can improve their knowledge and confidence in their practice. Researchers in the UK developed an evidence-based training programme consisting of a 10–12 h self-study breastfeeding workbook, a DVD teaching the two essential breastfeeding support skills of P & A and HE and used the Coventry University Breastfeeding Assessment tool (CUBA) to meet the requirements of UK UNICEF BFI accreditation standards in England [27–30]. Both have been tested with 322 midwifery and nursing clinicians in two maternity and community services in the West Midlands of England [30].The current research study is unique in China, using an objective method of assessment and a training DVD that has already been found to be effective the UK.

The current study aimed to test if the short DVD training programme is effective in improving Chinese clinicians’ knowledge and confidence in the specific skills of P & A and HE skills compared to controls not given the training but conducting the same repeated assessments of knowledge and confidence.

## Methods

### Intervention

The breastfeeding training DVD developed by the researchers in England was the training intervention for healthcare professionals in the study. It covers four educational sessions, including optimal P & A, the process of a satisfying feed shown by a baby of three months, hand expression demonstrated by a mother, and baby-led feeding demonstrated by an experienced baby of ten months. The total length of the DVD is 14 min 32 s [29]. The Mandarin script of the breastfeeding trainers’ voice-over explaining the learning points of the DVD was developed by the lead author based on the English script version, and agreed with two breastfeeding experts and one professional translator. Two staff in Zhejiang University of Media and Communications recorded and produced the Mandarin version of the breastfeeding training DVD according to the Mandarin script.

### Design

The study tests the effect of the Intervention Group (IG) watching the Breastfeeding DVD designed to educate clinicians to facilitate the development of knowledge and confidence in the skills of supporting mothers in P & A and HE compared to a Control Group (CG) of clinicians who watch a DVD about vaginal birth of similar duration on clinicians’ total knowledge of PA and HE (primary outcome) and confidence in P & A and HE (secondary outcomes) in a randomised controlled trial. The baseline measures were administered immediately before watching the DVD, and post intervention measures were taken immediately after watching the DVD. A power analysis was carried out to determine the required sample size in the study based on the a result of a pilot study among Chinese professionals conducted by the lead author [31]. The observed effect size was large (d = 1.05) for the t-test on change in total knowledge scores (primary outcome) between pre- and post-DVD. G*Power was used to calculate the required sample size to detect a condition by time interaction in ANOVA with a medium-to-large effect size (*F* = 0.30) [32]. In order to achieve 90% power, with significance level set at 5%, a total of 119 participants were required. We sought to invite approximately 200 participants to allow for 40% attrition.

### Setting

The study was conducted in Zhejiang Province, China. Participants were recruited from those clinicians about to attend a breastfeeding training course. They are all from three hospitals, including Women’s Hospital, School of Medicine, Zhejiang University, Kaihua county maternal and child health care hospital, and Kaihua county general hospital. The Women’s Hospital, School of Medicine, Zhejiang University is the largest and highest level teaching tertiary hospital for maternal health care in Zhejiang Province. The other two hospitals are secondary county hospitals in Kaihua county. All three hospitals are accredited as BFHI hospitals.

### Sample

Qualified health professionals (doctors, nurses and midwives) in Zhejiang Province who work with breastfeeding women were included. Staff who were not directly caring for breastfeeding mothers or volunteer for supporting breastfeeding mothers were excluded.

### Procedure

**Fig. 1:**
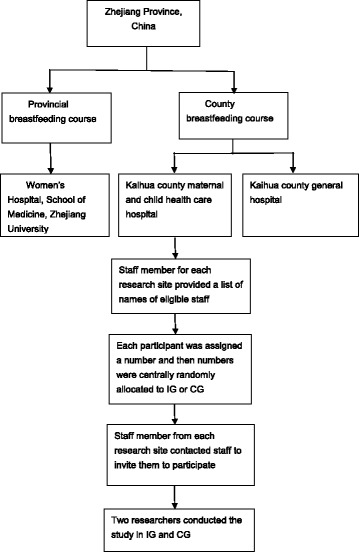
Recruitment procedure

The study was conducted before two breastfeeding training courses during March and June 2014. The researcher obtained the lists of names of all the participants who would take part in the provincial and county breastfeeding training courses. There was a staff member in each hospital who co-ordinated the study and emailed potential participants with an invitation to take part in the study. Those professionals who had no email address or did not reply to the invitation email were invited by telephone. The reasons for declining the invitation were recorded by the staff member. Participants who met the eligibility criteria and agreed to participate in each hospital were randomly allocated by a researcher blind to the two conditions to an intervention group (IG) and a control group (CG) using SPSS software by a computer professional in Zhejiang University. Randomisation was for the whole sample not by site. See Fig. 1.

All the participants received a sealed envelope marked Group 1 (IG) or Group 2 (CG), in which there was information for participants about the study, consent form, pre-DVD training questionnaire and post-DVD training questionnaire, both with a coded number sequence. Participants were not aware until they saw the DVD which group they were randomly allocated to in order to minimize disruption to the training courses, and they were informed they could withdraw consent at any time. The two groups were allocated to different rooms at the same time point and separate researchers explained the aims, methods and use of their data in line with the written information provided, and supervised the signing of consent forms. Pre-DVD training assessments (baseline questionnaire) were carried out for all participants before they watched the DVD. The baseline questionnaires were collected by the researchers immediately after being completed. Any one who withdrew consent to participate was asked to leave the room and wait for the training course. Next, the participants in the intervention group watched the Mandarin version of the breastfeeding training DVD. The control group watched a DVD in Mandarin unrelated to breasteeding about vaginal delivery. Both DVDs are of about 15 min. After watching their respective DVDs, participants in both intervention and control groups completed the post DVD training assessments.

### Measures

#### Knowledge

The level of knowledge of breastfeeding in P & Aand HE was assessed using a Mandarin version of mini-CUBA based on the Coventry University Breastfeeding Assessment (CUBA) used in a previously published study [29]. The items of mini-CUBA were multiple-choice questions with only one answer correct of four options, which had been agreed by a panel of three breastfeeding experts. There were six items where a correct answer is scored as 1 of P & A knowledge (range 0–6) and six items of HE knowledge (range 0–6). A higher score indicated a higher knowledge level.

#### Confidence

The confidence of healthcare professionals in skills for supporting breastfeeding mothers was measured using the Mandarin version of Coventry University Breastfeeding Support Self-Efficacy Scale (CU-BSSES) [29]. It measures six items of self-efficacy in supporting P & A and two items on teaching HE, using a 10-point Likert scale (1 = not at all confident; 10 = completely confident). CU-BSSES was used to assess the breastfeeding confidence among healthcare professionals in supporting the two skills of P & A and HE. Cronbach alpha coefficient value of 0.95 in this sample suggests a good internal reliability of CU-BSSES for the sub scale and total scale scores.

#### Job specific survey items

There were two personal and six job specific items: gender, age, work setting, job type, job title, years of qualification, working length with breastfeeding mothers, previous breastfeeding training. This was administered only at the baseline, along with knowledge and confidence measures. The post-training questionnaire comprised only the knowledge and confidence measures. The knowledge assessments of P & A and HE in the pre- and post-training questionnaires were the same. The change in scores of knowledge regarding the two skills of P & A and HE in pre- and post-training questionnaires were the primary outcome to assess the effectiveness of the breastfeeding training DVD.

### Analysis

Epidata (3.1) was used for double entry of data and the accuracy of entries was checked. Statistical Package for Social Science (SPSS version 22. 0) was used to analyse the data and the level of statistical significance was set at *p* < 0.05. Cases were excluded if they were missing data on any variable included in the specific analysis. For statistically significant groups, the strength of association was assessed by effect size to provide the magnitude of the differences between groups [33, 34]. Cronbach’s alpha coefficient was examined and reported the internal consistency for the Breastfeeding Support Self-Efficacy Scale. Chi-square tests compared the differences of each personal and job variable between the intervention and control groups. Mixed 2-way analysis of variance (ANOVA) were conducted to explore the interaction between group and time (pre-post DVD) for each dependent variable (PA knowledge, PA confidence, HE knowledge and HE confidence). Independent-Samples T Test was used to examine the differences in the mean score of knowledge and confidence between the two groups, and before and after viewing the DVD. The changes in total score of knowledge and confidence in the two skills in the intervention group by personal and job type variables was analysed by mixed ANOVA.

## Results

### Characteristics of participants

**Fig. 2:**
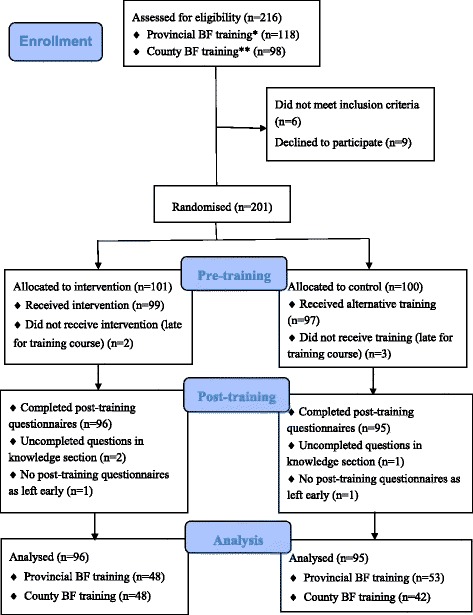
RCT Flow Diagram. *Participants in Provincial BF training were from Women’s Hospital, School of Medicine, Zhejiang University. ** Participants in County BF training were from Kaihua county maternal and child health care hospital and Kaihua county general hospital

Between March and June 2014, a total of 216 names were provided by the staff members at the research sites, including118 professionals participating provincial breastfeeding training course and 98 professionals in county training course. Six professionals were not eligible and were excluded as they worked in an administrative department. Nine staff declined to participate before the study with two staff stating they were not interested, and seven staff said they did not have time to participate in the study. The remaining 201 eligible staff were invited to take part in the study. Five professionals who were late for the breastfeeding training course did not participate in the study with 2 staff of IG and 3 staff of CG. Two clinicians did not complete the post-training questionnaires as they left early. Three post-training questionnaires were excluded during analysis due to half or more uncompleted items. The final sample included 191 healthcare professionals who watched the allocated DVD and completed the pre- and post-DVD training questionnaires: 96 in the IG and 95 in CG. The response rate was 91% (See Fig. 2).

The 191 participants covered a spread of professions, including 91 (47.6%) nurses, 39 (20.4%) midwives, 61 (32.0%) doctors. Most participants were female except five male doctors, as almost all professionals who work with breastfeeding mothers in China are female. These participants were diverse in age, work setting, job title, job type, length of time working with breastfeeding mothers, years since obtaining their professional qualification and previous breastfeeding training. The mean age of the participants was 33.51 years (SD = 8.17), however there was missing data for 51 cases for age. There were 101 (52.9%) working in provincial hospitals and 90 (47.1%) worked in county hospitals. There were 94 (49.2%) participants that had achieved mid-grade or higher titles and 97 (50.8%) participants had primary junior titles, i.e. those who were in their first post qualification job role). Furthermore, 90 (47.1%) participants had received their qualifications more than 5 years ago, and 101 (52.9%) participants less than 5 years. Some 132 (69.1%) participants had worked with breastfeeding mothers for more than two years, and 75 (39.3%) participants had worked for more than ten years. Of the study sample, 132 (69.5%) participants had had previous breastfeeding training and 58 (30.5%) participants had had no breastfeeding training prior to the training course. There were no significant differences on any of these variables between the two groups. (See Table 1).

**Table 1:** Characteristics of the professionals participating in the evaluation of the breastfeeding training DVD

Characteristics		IG *n* = 96 (%)	CG *n* = 95 (%)	X^2^ and *p*-value
Age	<30 years	22 (22.9)	24 (25.3)	3.84 *p =* 0.15
	30–39 years	35 (36.5)	26 (27.4)	
	>39 years Missing	12 (12.5) 27 (28.1)	21(22.1) 24(25.3)	
Working hospital	Provincial hospital	48 (50)	53 (55.8)	0.01 *p =* 0.92
	County hospital	48(50)	42(44.2)	
Job type	Nurse	46 (47.9)	45 (47.4)	0.38 *p =* 0.83
	Midwife	32 (33.3)	29 (30.5)	
	Doctor	18 (18.8)	21 (22.1)	
Job title	Senior or mid-grade	48 (50.0)	46 (48.5)	0.05 *p =* 0.83
	Junior grade	48 (50.0)	49 (51.5)	
Years since qualification	≤ 5 years	43 (44.8)	47 (49.5)	0.53 *p =* 0.47
	> 5 years	53 (55.2)	48 (50.5)	
Year of working with breastfeeding mothers	≤ 2 years	23 (24.0)	30 (31.6)	0.44 *p =* 0.80
	3–10 years	33 (34.4)	24 (25.3)	
	> 10 years	36 (37.5)	39 (41.1)	
	Missing	4(4.2)	2(2.1)	
Previous training on breastfeeding	Yes	67 (69.8)	65 (68.4)	0.01 *p =* 0.92
	No	29 (30.2)	29 (30.5)	
	Missing	0(0.0)	1(1.1)	

*IG* Intervention group

*CG* Control group

### Pre DVD knowledge and confidence scores in IG and CG

At pre DVD baseline, there was no statistical difference in P & A, HE and total knowledge score between IG and CG. There was missing data from 48 participants on the confidence measure at either pre or post -test, so confidence analyses were restricted to 143 cases. At pre-test there was missing data for 25/96 participants in the IG, and 19/95 in the CG, in the post-test there was missing data for 4/96 in the IG and 14/95 in the CG. The total confidence score and P & A confidence score at baseline showed no significant difference between IG and CG. However, the HE confidence score at baseline was significantly higher in the CG compared with IG. This data is available from the authors.Therefore change scores on knowledge and confidence are used in further analyses.

### Knowledge scores changes between pre- and post-DVD in IG and CG

Using ANOVA (Group x Time) there was significant interaction between group and time in total knowledge score [F(1,188) = 92.11, *p* < 0.001, η^2^ = .33.], P & A knowledge score [F(1,189) = 48.02, *p* < 0.001, η^2^ = .20] and HE knowledge score [F(1,189) = 48.65, *p* < 0.001, η^2^ = .21]. T-tests showed that in the IG there was a significant increase in total knowledge, P & A knowledge and HE knowledge after completing the DVD training, with large effect sizes (see Table 2). However, there was no significant change in total knowledge PA knowledge or HE knowledge control group (Table 2).The η^2^ values (> 0.4) indicate large increases in knowledge in the IG group.

**Table 2:** Comparison of the differences in knowledge scores between pre- and post-DVD in intervention group and control group

Items	IG (*n* = 96)			CG (*n* = 95)		
	Pre- DVD mean (SD)	Post- DVD mean (SD)	*t test*	Pre- DVD mean (SD)	Post- DVD mean (SD)	*t test*
P & A (range 0–6)	2.73 (1.41)	4.04 (1.16)	t (95) = −8.29 *p* < 0.01	2.86 (1.37)	2.83 (1.19)	t (94) = 0.28 *p =* 0.78
HE (range 0–6)	2.66 (1.09)	3.70 (1.00)	t (95) = −8.13 *p* < 0.01	2.81 (1.13)	2.73 (1.19)	t (94) = 0.86 *p* = 0.39
Total (range 0–12)	5.39 (2.03)	7.74 (1.71)	t (95) = −10.95 *p* < 0.01	5.67 (1.70)	5.56 (1.62)	t (94) = 0.85 *p* = 0.40

### Confidence scores changes between pre- and post-DVD in IG and CG

There were 135 participant completed the pre-post confidence assessment. The participants’ characteristics of those with complete data were compared with those with missing data and no difference observed. There was a significant interaction between group and time (pre-post DVD) in total confidence score [F(1,133) = 17.92, *p* < 0.001, η^2^ = 0.26], P & A confidence score F(1,133) = 15.66, *p* < 0.001, η^2^ = 0.26] and HE confidence score [F(1,135) = 13.90, *p* < 0.001, η^2^ = 0.20]. T-tests revealed a significant increase in the P & A confidence scores was found post-DVD compared to pre-DVD in the IG, but not the CG (see Table 3). The Eta squared value (> 0.2) indicate large increases in confidence in the IG group.

**Table 3:** Comparison of differences in confidence scores between pre- and post-DVD in intervention group and control group

Items	IG (*n* = 69)			CG (*n* = 66)		
	Pre- DVD mean (SD)	Post- DVD mean (SD)	*p*-value	Pre- DVD mean (SD)	Post- DVD Mean (SD)	*p*-value
P & A (range 0–60)	49.93 (8.48)	53.90 (6.93)	t (68) = −4.79 *p* < 0.01	50.68 (8.62)	50.82 (8.89)	t (65) = −0.36 *p =* 0.72
HE (range 0–20)	16.57 (3.29)	17.91 (2.51)	t (68) = −4.12 *p* < 0.01	17.59 (2.85)	17.49 (2.77)	t (67) = 0.49 *p* = 0.62
Total (range 0–80)	66.49 (11.27)	71.81 (9.33)	t (68) = −4.92 *p* < 0.01	68.33 (11.08)	68.35 (11.40)	t (65) = −0.25 *p* = 0.98

Cases were deleted where there was missing data on the dependent variable

### Sub group analyses on change in knowledge scores of the intervention group

Table 4 shows the change in total knowledge scores in the IG. All the sub-groups (within-subjects) achieved significantly increased total knowledge scores post-DVD compared to pre-DVD. A significantly higher increase in total knowledge score was seen among the participants working in county hospitals or less specialised (lower level) hospitals compared to participants in provincial or municipal hospitals. There was also a significant sub group effect (interaction) for job type. Inspecting the mean scores shows doctors achieved a greater increase in total knowledge score compared to midwives and nurses. However, the post hoc showed no significant differences in increased total knowledge scores between doctor and midwife, or doctor and nurse, or nurse and midwife. There were no significant differences in the change in knowledge scores for other sub-group variables, including age, seniority of job (job title), length of time working with breastfeeding mothers, years since obtaining professional qualification and previous breastfeeding training.

**Table 4:** Change in the total knowledge scores in sub-group variables of intervention group between pre- and post-training

Items		Number	Pre-DVD mean (SD) (range 0–12)	Post-DVD mean (SD) (range 0–12)	ANOVA (interaction effect)	ANOVA (within-subject effect)
Age	<30 years	22	5.55 (1.57)	7.18 (1.40)	F(2,66) = 0.80 *p =* 0.45	F(1,66) = 64.33 *p* < 0.01
	30–39 years	35	5.66 (2.16)	7.94 (1.73)		
	>39 years	12	5.25 (2.53)	7.33 (1.88)		
Working hospital	Provincial or municipal hospital	35	6.31 (1.81)	7.94 (1.64)	F(1,94) = 6.94 *p* = 0.01	F(1,94) = 102.95 *p* < 0.01
	County hospital or below	61	4.85 (1.97)	7.62 (1.74)		
Job type	Nurse	46	5.57 (1.97)	7.67 (1.52)	F(2,93) =3.06 *p =* 0.05	F(1,93) = 102.83 *p* < 0.01
	Midwife	18	6.28 (2.05)	8.00 (2.17)		
	Doctor	32	4.63 (1.90)	7.69 (1.71)		
Job title	Senior or mid-grade	48	5.71 (2.25)	8.06 (1.93)	F(1,94) =0.00 *p* = 1.00	F(1,94) = 118.52 *p* < 0.01
	Primary	48	5.06 (1.76)	7.42 (1.40)		
Years since qualification	≤ 5 years	42	5.48 (1.71)	7.43 (1.53)	F(1,93) = 2.67 *p* = 0.11	F(1,93) = 113.15 *p* < 0.01
	> 5 years	53	5.30 (2.28)	7.96 (1.82)		
Years working with BF mothers	≤2 years	23	5.39 (1.85)	7.22 (1.57)	F(2,89) = 0.93 *p =* 0.40	F(1,89) = 100.48 *p* < 0.01
	3–10 years	33	5.09 (1.93)	7.70 (1.69)		
	> 10 years	36	5.81 (2.27)	8.14 (1.81)		
Previous training on breastfeeding	Yes	67	5.87 (1.95)	7.96 (1.75)	F(1,94) = 3.59 *p =* 0.06	F(1,94) = 119.62 *p* < 0.01
	No	29	4.28 (1.81)	7.24 (1.53)		

Cases were deleted where there was missing data on the independent or dependent variable

Table 5 shows the impact of the breastfeeding training DVD on the change of mean confidence scores in the sub-groups of the intervention group from pre-training to post-training. Cases were deleted where data was missing on one or more variable. All the sub-groups in the IG obtained significantly increased total confidence scores post-DVD compared to pre-DVD. No significant differences in mean confidence scores were found for any sub-groups of personal and job-related variables. (See Table5).

**Table 5:** Change in total confidence scores in sub-groups of intervention group between pre- and post-training

Items		Number	Pre-DVD mean (SD) (range 0–80)	Post-DVD mean (SD) (range 0–80)	ANOVA (interaction effect)	ANOVA (within-subject effect)
Age	<30	14	68.43 (11.01)	69.36 (9.10)	F(2,48) =1.25 *p* = 0.30	F(1,48) =23.63 *p* < 0.01
	30–39	30	69.63 (9.35)	72.73 (7.33)		
	>39	7	69.00 (13.42)	73.43 (8.00)		
Working hospital	Provincial /municipal hospital	30	68.67 (10.38)	72.10 (8.58)	F(1,67) =2.39 *p* = 0.13	F(1,67) =22.31 *p* < 0.01
	County hospital	39	64.82 (11.76)	71.59 (9.97)		
Job type	Nurse	35	68.57 (9.73)	71.43 (8.50)	F(2,66) =2.97 *p* = 0.06	F(1,66) =29.91 *p* < 0.01
	Midwife	15	67.00 (11.09)	73.93 (7.27)		
	Doctor	19	62.26 (13.32)	70.84 (12.09)		
Job title	Senior or mid-grade	32	68.35 (11.11)	73.19 (7.49)	F(1,67) =0.23 *P =* 0.64	F(1,67) =24.12 *p* < 0.01
	Primary	37	64.34 (11.24)	70.22 (11.00)		
Years since qualification	≤ 5 years	28	65.89 (11.45)	71.11 (7.63)	F(1,66) =0.02 *p* = 0.89	F(1,66) =22.97 *p* < 0.01
	> 5 years	40	66.77 (11.38)	72.30 (10.53)		
Years working with BF^a^ mothers	≤2 years	14	60.50 (12.79)	68.43 (8.29)	F(2,64) =0.89 *p* = 0.42	F(1,64) =24.43 *p* < 0.01
	3–10 years	24	67.21 (10.26)	72.46 (7.35)		
	> 10 years	29	68.41 (10.81)	72.41 (11.17)		
Previous training on BF	Yes	51	68.47 (10.01)	73.08 (7.34)	F(1,67) =1.23 *p =* 0.27	F(1,67) =23.58 *p* < 0.01
	No	18	60.89 (12.97)	68.22 (12.39)		

^a^*BF* Breastfeeding

Cases were deleted where there was missing data on the dependent variable

## Discussion

This is the first randomised controlled trial of the effectiveness of a DVD training programme on improving breastfeeding knowledge and confidence in practice of the two key skills of P & A and HE among professionals in China. We were able to show that the effect is not simply attributable to practise on the knowledge assessment and confidence ratings, since there was no test and retest effect in the CG. Second, the increases were statistically significant in the IG. Further, the improvements were shown for all types of staff when analysed by personal and job variables.

In conducting the RCT, every effort was made to prevent known sources of bias. Randomisation was conducted independently of the researchers, trainers and participants. Allocation concealment was maintained so participants were blind to allocation until after the baseline data was collected. Analyses showed that groups were balanced for personal and job variables which might have affected baseline levels of knowledge. Missing data was evenly distributed between control and intervention groups. No significant differences in the mean score of knowledge and confidence were found between groups in two areas of P & A, except that the control group had higher baseline confidence in HE than the IG group before they watched a DVD. Participants came from a range of maternity services in Zhejiang Province, and a range of clinical professions, with differing lengths of experience of working with breastfeeding mothers, time since gaining their qualifications and levels of prior breastfeeding training. Thus, the sample was broadly representative of the healthcare professionals supporting breastfeeding mothers in Zhejiang Province, and with a high response rate.

### Knowledge

The study showed a large range of knowledge scores and poor knowledge level at baseline. The mean scores were less than half of the total possible correct scores at baseline among participants in total. Although clinical staff in BFHI accredited hospitals are expected to be trained in breastfeeding support as soon as they are employed in a clinical role with mothers, this study suggests many women will be exposed to clinical staff with insufficient knowledge of these essential skills. Such results are perhaps to be expected, given the acknowledged shortfalls in implememtaion of BFHI in China referred to above [24, 35].

The study indicates the new DVD training method is effective in improving professionals’ knowledge in each area of P & A, HE. Improvements in mean knowledge scores were not moderated by age, time working with breastfeeding mothers, job type, years since qualification, seniority (job title) and prior to breastfeeding training experience.This finding is similar to a study which assessed breastfeeding knowledge among 51 clinicians in a neonatal unit in England using a similar multiple choice test of knowledge, where personal and job variables similar to those in this study were not associated with differences in objectively assessed knowledge [36].

Further sub-group analysis shows a significantly higher mean knowledge score was found in specialist tertiary hospitals than in the less specialist local hospitals before DVD training. This is consistent with the findings from the study by Wang and colleagues who reported the area of maternal service to be one of the crucial factors influencing breastfeeding duration due to the different support services provided by professionals in different settings, based on 47,843 mother-infant pairs under two years of age in ten provinces of China [37]. In our study, significantly improved scores were achieved in both types of hospital, showing that a short training intervention may contribute to reducing this variation in the provision of breastfeeding support.

Other sources of unequal provision arise from the large economic differences between provinces. The eastern coast of China is more developed and has more high quality education in medicine than those in rural areas [38]. If the results of this study can be achieved in these less prosperous areas, it may help to reduce the inequalities in breastfeeding training between low-income regions and richer areas of China. Further studies could test whether the breastfeeding DVD training achieves the same results when offered for home self study, enabling more clinicians to meet their personal learning objectives.

### Confidence in practice

For those who completed the confidence measure, significantly increased total confidence scores were found in the intervention group between pre- and post-training, but not in the control group. Also, there were no significant differences in the change of total confidence scores in each sub-group between pre- and post-training. Our results are limited by the 48 participants who did not complete one of the measures of confidence. Neverthelss, these findings are consistent with other studies that show breastfeeding education interventions for clinicians significantly increase their confidence in practice [39–42]. Downie, Rakic and Juliff reported that the increased confidence among nurses and midwives continued after completing the intervention Lactation Advisor Program (LAP) in Australial [39]. Similarly, in England, midwives and health visitors had increased confidence after BFHI training, which they reported enabled them to be more confident in supporting breastfeeding mothers [43]. The authors of a synthesis of breastfeeding training interventions concluded that proficient skills and increased confidence in helping breastfeeding mothers among healthcare professionals can be improved by training interventions, and this may improve rates of duration and exclusivity of breastfeeding [25]. Further study in China is require to examine the effect of the increased confidence on improving professionals’ practice in supporting mothers’ breastfeeding skills, thereby increasing the breastfeeding duration and rate of exclusive breastfeeding.

### Limitations

The retest of the breastfeeding knowledge in the study was conducted immediately after both groups were exposed to the DVD, so only a short term effect has been measured. And no measures have been made in this study of changes in clinical practice and in breastfeeding rates. The large amount of missing data in the confidence scales suggests that the measure may not be understood, and needs further methodological development for use in the Chinese population.

## Conclusion

Overall, this study has shown that the low levels of knowledge in positioning and attachment and hand expression of a range of clinical staff who are regularly engaged in supporting breastfeeding mothers in Chinese hospitals with BFHI accreditation can be addressed by a short training DVD. This method may be suited to addressing training needs more widely in China due to its effectiveness on knowledge, and the feasibility and low cost to staff time for large numbers of staff. However, the effectiveness of the DVD training on improving professionals’ knowledge and confidence in practice the longer term requires further study, and the translation of knowledgeable and confident practice into the support that mothers need to improve breastfeeding outcomes should be further examined in the future.

## Abbreviations

BFHI: Baby Friendly Hospital Initiative
CG: Control group
CUBA: Coventry University Breastfeeding Assessment tool
CU-BSSES: Coventry University Breastfeeding Support Self-Efficacy Scale
FIL: Feedback inhibitor of lactation
HE: Hand Expression
IG: Intervention group
NHFPC: National Health and Family Planning Commission
NPA: National Programme of Action for Children’s Development
P & A: Positioning and Attachment
WHO: World Health Organization
